# Supplementary material for: Hepatitis B screening and vaccination status of healthcare providers in Wakiso district, Uganda
Source: PLoS One. 2020 Jul 9;15(7):e0235470. doi: 10.1371/journal.pone.0235470 (PMC7347299; doi:10.1371/journal.pone.0235470)
Supplement: S1 Appendix — (DOCX) [file pone.0235470.s001.docx]

| **Appendix 1. STUDY TITTLE:** HEALTH CARE PROVIDERS’ HEPATITIS B VACCINATION STATUS AND THEIR LEVEL OF KNOWLEDGE ATTITUDE AND PRACTICE TOWARDS PROPHYLACTIC MANAGEMENT OF HBV: A CROSSECTIONAL SURVEY IN WAKISO DISTRICT. | | | |
| --- | --- | --- | --- |
| **IDENTIFICATION INFORMATION** | | | |
| **Date of interview** | |  | |
| **Questionnaire No.** | |  | |
| **Initials of interviewer** | |  | |
| Level of Health Facility | | 1. Hospital 2. Health Centre IV 3. Health Centre III 4. Health Centre II | |
| GPS coordinates | |  | |
| Ownership of Health Facility | | 1. Public 2. Private 3. PNFP | |
| Location of Health facility | | 1. Urban 2. Rural | |
| Name of Health Sub district | | 1. Busiro North 2. Busiro East 3. Busiro south 4. Kyadondo North 5. Kyadondo south 6. Kyadondo East 7. Entebbe municipality | |
| **Background characteristics** | | | |
| **No.** | **Question** | **Response** | **SKIP** |
|  | Sex | 1. Male 2. Female |  |
|  | Age (complete number of years) |  |  |
|  | Marital status | 1. Single (never married) 2. Married/cohabiting 3. Separated/divorced 4. Widowed |  |
|  | Cadre | 1. Medical doctor 2. Clinical officer 3. Laboratory technician/ technologist 4. Laboratory assistant 5. Registered nurse 6. Enrolled nurse 7. Registered midwife 8. Enrolled midwife 9. Nursing assistant 10. Other (please specify) |  |
|  | Department of work | 1. OPD 2. IPD 3. Maternity 4. Theatre 5. Laboratory 6. Others (specify) |  |
|  | For how long have you been practicing? (Duration in complete years) |  |  |
| **KNOWLEDGE ON TRANSMISSION OF HEPATITIS B INFECTION** | | | |
|  | Have you ever had of hepatitis B? | 1. Yes 2. No |  |
|  | What causes hepatitis B? | 1. Virus 2. Bacteria 3. Protozoa 4. Fungi |  |
|  | How is hepatitis B transmitted? (Tick all that apply) | 1. Sexual contact 2. Mother to child transmission 3. Blood transfusion 4. Sharing of sharps 5. Other (specify) |  |
|  | Do you think hepatitis B can be transmitted by carriers? | 1. Yes 2. No |  |
|  | Do you think you are at risk of hepatitis B infection? | 1. Yes 2. No | If No, go to Qn 12 |
|  | If yes, to what extent are you at risk? | 1. Low 2. Moderate 3. High |  |
|  | Which other groups are at risk of contracting hepatitis B? (Tick all that apply) | 1. Female sex workers 2. Men who have sex with men 3. Injecting drug users 4. Long truckers 5. Others (specify) |  |
|  | What increases the risk to hepatitis B infection in a health care setting? | 1. Sharing sharps 2. Unsafe injection practices 3. Unsafe waste handling 4. Blood transfusion 5. Other (specify) |  |
|  | Which body organ is mainly affected by hepatitis B infection? | 1. Liver 2. Kidney 3. Brain 4. Other |  |
|  | What are the symptoms of hepatitis B? | 1. Fever 2. Vomiting 3. Jaundice 4. Abdominal discomfort 5. Clay coloured stools 6. Convulsions 7. Other (specify) |  |
|  | What are the complications associated with hepatitis B infection? | 1. Acute hepatitis 2. Liver cirrhosis 3. Liver cancer 4. Anaemia 5. Hepatic encephalopathy |  |
|  | Is Hepatitis B treatable? | 1. Yes 2. No |  |
|  | If yes, how is it treated? | 1. Use anti-retroviral drugs 2. Use supportive treatment 3. Other (Specify) |  |
| **Knowledge on prevention of Hepatitis B** | | | |
|  | Is hepatitis B preventable? | 1. Yes 2. No | **If yes go to Qn 21.** |
|  | How can hepatitis B be prevented in a health care setting? | 1. Screening of blood before transfusion 2. Vaccination against HBV 3. Use of protective gears like gloves 4. Safe waste management 5. Safe injection practices (including minimum use of syringes) 6. Other (specify) |  |
|  | Do you think hepatitis B has a test? | 1. Yes 2. No |  |
|  | Have you ever taken a hepatitis B test? | 1. Yes 2. No | **If no, skip to the next section** |
|  | If yes, how long ago? |  |  |
|  | If you don’t mind, can you share with me your hepatitis B status? |  |  |
| **Hepatitis B vaccination** | | | |
|  | Have you ever heard about hepatitis B vaccination? | 1. Yes 2. No |  |
|  | How effective do you think hepatitis B vaccination is in protecting someone against hepatitis B virus infection? | 1. Not effective 2. Slightly effective 3. Very effective 4. I don’t know |  |
|  | Have you ever received hepatitis B vaccination? | 1. Yes  2. No | **If Yes, skip to Qn. 28.** |
|  | Why have you not received hepatitis B vaccination? (Multiple responses allowed) | 1. I am not aware of hepatitis B vaccination 2. I do not know where to go and receive it 3. I don’t have time 4. It is expensive 5. I don’t see the need 6. I am afraid of contracting the virus from the vaccine 7. Others (Specify) |  |
|  | On which part of the body was the vaccine administered? | 1. Thigh 2. Left arm 3. Right arm 4. Mouth 5. Other (specify) |  |
|  | If your answer to question 26 is ‘Yes’, how many doses of hepatitis B vaccine have you received? | 1. 1 dose 2. 2 doses 3. 3 doses 4. More than 3 doses |  |
|  | When did you receive the last dose of hepatitis B vaccine? | 1. Less than one month ago 2. 1-3 months 3. 4-6 months ago 4. More than 6 months ago |  |
|  | What do you think is the recommended full dose of hepatitis B vaccine? | 1. 1 dose 2. 2 doses 3. 3 or more doses 4. I don’t know |  |
|  | How long does a full dose of hepatitis B vaccine protect someone? | 1. Less than 1 year 2. 1- 5 years 3. 6-10 years 4. 11-19 years 5. 20 years and more 6. I don’t know |  |
|  | Have you ever heard about a hepatitis B immune response test? | 1. Yes 2. No |  |
|  | Have you ever done a hepatitis B immune response test? | 1. Yes 2. No | **If yes, skip the next question** |
|  | If no, why? | 1. Have never heard about it? 2. It is costly 3. Feared the test 4. Other (Specify) |  |
|  | Have you ever heard about PEP for hepatitis B? | 1. Yes 2. No |  |
|  | Where did you hear about PEP for hepatitis B? | 1. Yes 2. No |  |
|  | Have you ever been in a training on PEP? | 1. Yes 2. No |  |
|  | **Attitude towards Hepatitis B in Health care settings** | |  |
|  | Do you think your job puts you at a higher risk of acquiring hepatitis B? | 1. Yes 2. No |  |
|  | Do you think you need to be protected from Hepatitis B infection? | 1. Yes 2. No |  |
|  | Do you think all patients should be tested for hepatitis B prior to being given treatment? | 1. Yes 2. No |  |
|  | Do you think a health care provider can infect patients with Hepatitis B infection? | 1. Yes 2. No |  |
|  | Do you think it is necessary to receive hepatitis B vaccine? | 1. Yes 2. No |  |
|  | Do you think the hepatitis B vaccine is safe? | 1. Yes 2. No |  |
|  | Do you think the hepatitis B vaccine is effective? | 1. Yes 2. No |  |
|  | Do you think following infection control guidelines will protect from being infected with HBV at work? | 1. Yes 2. No |  |
|  | Have your children received Hepatitis B Vaccine? | 1. Yes 2. No 3. Not applicable (No children) |  |
|  | Do you think the hepatitis B vaccine can be used for post exposure prophylaxis? | 1. Yes 2. No |  |
|  | **Practice related to Hepatitis B prevention** | |  |
|  | Have you ever had a needle stick injury? | 1. Yes 2. No | **If No, skip to Qn 5.** |
|  | Did you report when this injury? | 1. Yes 2. No |  |
|  | Have you ever been screened for Hepatitis B? | 1. Yes 2. No |  |
|  | Do you ask for screening of blood before transfusion? | 1. Yes 2. No 3. Not applicable (Does not transfuse) |  |
|  | Do you ask for a new syringe before use? | 1. Yes 2. No |  |
|  | Have you ever participated in a healthrelated program on hepatitis B? | 1. Yes 2. No |  |
|  | Does hepatitis B have post exposure treatment? | 1. Yes 2. No | **If no, skip to qn. 39** |
|  | What is used as PEP against Hepatitis B infection? | 1. Hepatitis B vaccine 2. Hepatitis B Immune Globulin 3. Other (specify) 4. I don’t know |  |
|  | Have you ever heard about Hepatitis B Immune Globulin (HBIG)? | 1. Yes 2. No |  |
|  | Where is HBIG administered? | 1. Intramuscular 2. Oral 3. Intradermal 4. Other (specify) |  |
|  | Do you think HBIG provides long term protection against hepatitis B infection? | 1. Yes 2. No |  |
|  | What steps would you undertake if you or a colleague gets exposed to a blood borne infection (focus on hepatitis B) ***(tick all correct responses)***. | 1. Provide immediate care to the exposure site 2. Determine risk associated with exposure by fluid and type of exposure 3. Evaluate exposure source (test the status of the exposure source) 4. Evaluate the exposed person for their immune status to the infection 5. Give PEP 6. Follow up testing 7. Provide counselling 8. Other (specify) |  |

**END: Thank you for your time**
